# Getting evidence to travel inside public systems: what organisational brokering capacities exist for evidence-based policy?

**DOI:** 10.1186/s12961-018-0393-y

**Published:** 2018-12-17

**Authors:** Pernelle Smits, Jean-Louis Denis, Johanne Préval, Evert Lindquist, Miguel Aguirre

**Affiliations:** 1 Université Laval, Pavillon Palasis-Prince, 2325 Rue de la Terrasse, Ville de Québec, QC G1V 0A6 Canada; 2 School of Public Health, Université de Montréal, Centre de Recherche du CHUM, 900, rue Saint-Denis, Pavillon R, Montréal, QC H2X 0A9 Canada; 3 ENAP École Nationale d’Administration Publique, 4750 Henri Julien, Montréal, QC H2T 3E5 Canada; 4 University of Victoria, 3800 Finnerty Road, Victoria, BC V8P 5C2 Canada

**Keywords:** Evidence-informed policy, knowledge broker, innovation broker, policy entrepreneur, policy broker, policy capacity, intersectoriality

## Abstract

**Background:**

Implementing research findings into healthcare policy is an enduring challenge made even more difficult when policies must be developed and implemented with the help and support of multiple ideas, agendas and actors taking part in determinants of health. Only looking at mechanisms to feed policy-makers with evidence or to interest researchers in the policy process will simply bring partial clues; implementing evidence-based policy also requires organisations to lead and to partner in the production and intake of scientific evidence from academics and practical evidence from one another.

**Main body:**

This Commentary argues for the need to better understand the capacities required by organisations to foster evidence-based policy in a dispersed environment. It proposes a framework of 11 brokering capacities for organisations involved in evidence-based policy. Eight of these capacities are informed by streams of research related to the roles of knowledge broker, innovation broker and policy broker. Three complementary brokering capacities are informed by our experience studying real-life evidence-based policies; these are capturing boundary knowledge, trending know-how on scientific and practical evidence-based policy, and conveying evidence outward.

**Conclusions:**

Previous guidelines on brokering capacities focused on the individual level more than on the organisational level. Beyond the individual capacities of managers, designers and implementers of new policies, there is a need to identify and assess the brokering capacities of organisations involved in evidence-based policy. The three specific organisational brokering capacities for evidence-based policy that we present offer a means for policy-makers and policy designers to reflect upon favourable environments for evidence-based policy. These capacities could also help administrators and implementation scholars to think about and develop measurements to assess the quality and readiness of organisations involved in evidence-based policy design.

## Main text

### Background

The evidence-based policy (EBP) or evidence-informed policy-making movement has gained wide interest over the last 40 years [1]. However, despite some policies being designed or adjusted based upon evidence, consensus remains that “[r]*esearchers and policy-makers are missing chances to turn research findings into lasting change*” [2].

New jobs and functions are among the solutions currently being explored in an effort to make the best decisions based on available knowledge, including knowledge brokers, innovation brokers and policy brokers, the latter also known as policy entrepreneurs [2]. Knowledge brokers are lauded for bridging the working environments of researchers (the knowledge producers) and decision-makers (the potential knowledge users). For example, in the private sector, structures such as innovation clusters play this role by linking researchers and industries from a determined field. In public administrations, institutions might play this brokerage role as well, fostering exchanges and linkages between researchers and public decision-makers. Put simply, a knowledge broker is an actor who provides scientific evidence to decision-makers in a timely and appropriate format, and feeds evidence into policy. An innovation broker is an actor who supports new technological or social ideas for commercial purposes and/or social betterment, transforming ideas into new strategies and scientific results into useable material for policies. Additionally, knowledge and innovation must be given direction, especially in the design of policies that operate in a dispersed environment; a policy-broker role is therefore also important. A policy broker is an actor who holds influence and ensures action towards the approval of ideas; they may be either a public actor (an insider) or a private actor (an outsider). In short, a policy broker makes things happen.

In acknowledging that better results could be achieved with what we already know, effort has been concentrated on bringing evidence into policy. This sound process for decision-making faces a few challenges such as switching managers’ mindsets away from managing based upon corridor talk or urgent responses to mediatised situations towards managing based upon evidence. The manager or their team needs to acquire specific expertise such as identifying evidence sources, appreciating the quality of evidence, transferring the results of studies to their context, and so forth.

To reinforce capacity to make good policy, scholars have already identified two important levers, namely individual competencies and organisational capacities*.* Individual competencies refer to one’s background, knowledge and experience, the practical skills of policy-making, and personal attributes. At the organisational level, capacities cover “*access to and use of information and evidence; personnel management and workforce development; consultation and communication; inter-departmental coordination and networking; implementation, monitoring, evaluation and review; strategic management and leadership; and institutional culture*” [3]. We focus on the organisational level and, in particular, the roles that an organisation will have to enact to reinforce EBP and what specific organisational capacities could be investigated by implementation scholars.

We argue that new organisational capacities need to be enacted for evidence to better travel within public administrations. We propose learning and highlighting the organisational capacities required for this to occur. We mobilise three streams of literature – concerning the roles of knowledge broker, innovation broker, and policy broker – and propose an analysis at the organisational level.

To begin, an illustrative story serves to highlight the need to better understand the capacities required by organisations to foster an EBP design.

### Main text

#### An illustrative story of difficult evidence circulation within public administration: the formulation of EBP

This illustrative story details an extreme situation of the circulation of evidence inside several public administrations. Resemblance to any specific policy is coincidental. Let us consider the formulation steps of an intersectoral policy in Canada, which we shall refer to as ‘policy AB’. Policy AB has governmental approval and media visibility. One ministry is in the driving seat, holds leadership, organises consultations and ensures the management of the formulation process. Policy AB is an interministerial policy, such Health in All Policies or the Policy on Well-being. It is explicitly stated that the intent of the policy is to move away from traditional and past strategies and actions. Let us consider the initial intent is to formulate the policy based on evidence, integrating determinants of health and known strategies to reinforce health-friendly policies in the economic sector, in the environmental sector, and so forth.

First, dozens of scientists are directly involved in gathering evidence, identifying the actions and proposing measures. Exchanges of evidence take place among scientists and among public servants participating in the formulation process of policy AB. Second, a wide consultation reaches all ministries for them to become acquainted with the evidence provided in step one and to co-filter some evidence-based measures based upon sectorial feasibility. At this step, the circulation of evidence takes place between ministries. Participants of step one may carry the evidence inside each ministry. Consider, for instance, professionals from the Ministry of Agriculture, who are updated on new strategies for health-friendly distribution and production procedures during policy AB discussions, and then share that information with public servants from their own ministry. Similarly, public servants from the Ministry of Environment and Ministry of Economy hear about such practices and reframe or extend their initial understanding and zone of action. In parallel, ministry-related actors transfer their sectorial knowledge to their sectorial peers, making sector-related, state-of-the-art knowledge in one field available to the entire group working on policy AB. Third, professionals in ministries and ministry directors meet to select and rank measures that were deemed socially, economically and politically viable. Lastly, some options are integrated into the final draft of policy AB, others are reformulated and some are deemed difficult to implement at that stage or face sector discomfort and opposition, eventually being transformed into research projects for further investigation.

Such a policy formulation process is a fair attempt to encourage EBP. Indeed, scientists bring evidence to the table. They interact with professionals from the various ministries during committee meetings held to present knowledge and make it understandable to all before a decision can be made on which measures to ultimately select. To a certain level, the final draft of policy AB integrates evidence.

Being intersectoral in nature, policy AB exacerbates the challenges related to the design of EBP, because it takes place in an environment where actors (individuals and organisations) have to collaborate to design a policy based on evidence but where they have no obligation to work together and do not share an understanding of the importance or quality of evidence. We can point to a few challenges that must be addressed in such a situation. For example, how is evidence gathered, considering each sector collects just bits and pieces? How is evidence integrated into the understanding of all participants, when each is accustomed to manipulating one set of quantitative, qualitative, economic or social-oriented data? How are traditions reconciled when one participant sees the gold standard of evidence sources in independent systematic reviews and the other sees it in a government-led commission report? How is the circulation of evidence ensured between participants with diverse mindsets (public and private sector, or professionals close to local implementation issues and managers close to strategy and political alignment)? A key role is played by the organisation in charge of bridging between the various actors and their representation of evidence, otherwise known as the broker role. We argue that the lead organisation for a policy design that wants to implement EBP, or that at a minimum wants to boost the EBP mindset among the public apparatus, would benefit from combining three roles, namely knowledge broker, innovation broker and policy broker.

#### The knowledge broker’s role in designing EBP: transfer, translate, transform and capture boundary knowledge

As promoted by the EBP movement, a knowledge broker is either an individual or an organisation with an explicit mandate to bridge evidence and decision-making [4] by passive transfer or more active actions. Generally speaking, a knowledge broker ensures functions such as strategic intelligence, translation of decision-makers’ needs into scientific enquiries, translation of evidence into the realm of policy-makers, seeking out ideas, and so forth.

The main knowledge-broker capacities, which we could term knowledge-broker know-how, can be summarised in the following manner: transferring information from a producer to a recipient, translating knowledge into understandable information and shared meaning, and transforming knowledge into adequate objects for interested actors or into new knowledge.

What does this look like for policy AB? The lead ministry ensures the transfer role when it gathers evidence from literature reviews with the help of scientists and presents that evidence to participants of the formulation process. It ensures the translation role via organised discussions around key messages from the literature with professionals from various ministries, trying to clarify newly collected evidence. It also ensures this translation role when it organises a systematic selection of the key messages based upon feasibility, political viability and other criteria cited above. The transformation role takes place when the collected evidence is framed into a summary sheet for each sector to be able to read targeted messages in a short time period, or when the lead ministry designs exercises from the collected evidence; for example, selecting and examining new public transport initiatives that would favour the health of all.

Considering EBP at the design stage calls for implementation scholars to reflect upon the following additional capacity: capturing boundary knowledge. What we mean is that designing a policy requires a collective vision among those involved, a vision whereby the policy will entail more than the sum of each individual participant’s knowledge and sometimes more than the various ministerial jurisdictions; this means filling in blanks between commonly established departmental or sectorial borders. Therefore, the broker will have to ascertain which areas are not known and not generally taken care of by the actors involved – regardless of whether those actors are private or public, departments in a single ministry, or sectors across ministries. In other words, the governance of an EBP cannot downplay the importance of stepping beyond individual and sectoral knowledge, seeking information at or beyond the boundaries of the individual’s or sector’s knowledge base, namely ‘boundary knowledge’.

We argue that a ministry and its team involved in the formulation of a governmental policy must consider capturing the information that falls outside these boundaries – between two chairs, so to speak – as well as any information that may seem contradictory at first glance. For example, for policy AB, each ministry already has its own field of action, and each department and team already have their own projects and programmes to run. Boundary knowledge can consist of topics highlighted by the literature but lagging in current sectorial developments (e.g. public collective accountability for policy AB), emerging topics that are outside the scope of participating ministries (e.g. the sexual health of elderly LGBTQ in residential housing), disruptive strategies that leave all ministries uncomfortable (e.g. strategies to fight paedophilia with the involvement of perpetrators), and so forth. A new policy would require collecting evidence outside the immediate established boundaries of existing projects and programmes. In addition to standard knowledge-broker capacities, capturing such boundary knowledge appears relevant to organisations fostering EBP.

#### The innovation broker’s role in designing EBP: articulating needs and demands, forming networks, managing innovation and trending know-how on EBP

It has long been known that innovations in and the content of new policies do not pop up ex nihilo but rather require well-planned and organised support to emerge. Actors taking charge of supporting innovation are known as innovation brokers [5]. An innovation broker is an “*organization that both acts in a liaison role between the sources of new ideas and the user of those ideas in innovation networks, and* [is] *also set up specifically to perform this broking role*” [5]. Innovation brokers act as liaisons between the sources of new ideas and the users of those ideas within innovation networks. They can assume the roles of mediator/arbitrator, sponsor/funds provider, filter/legitimator, technology broker, and finally resource/management provider. They manage ideas, new projects, funding and knowledge, build early networks and teams, and detect early trends, among other things. The innovation broker’s role changes throughout the lifetime of an innovation.

Overall, a great number of functions are attributed to innovation brokers, with authors proposing a set of functions [6]. A more aggregated categorisation is proposed around three basic functions, namely demand articulation (articulating innovation needs and corresponding demands), network formation (facilitating linkages between relevant actors), and innovation process management (enhancing alignment and learning of the multi-actor network). We will see in the next section on policy brokers that enabling collaborative networks is common to innovation brokers and policy brokers.

Let us go back to policy AB and see what the role of innovation broker would look like. In the formulation of policy AB, articulating demand takes place whenever the lead ministry seizes sectorial needs and turns them into opportunities to foster specific actions while trying to please all partnered ministries. For instance, when a ministry with a relatively small budget lacks visibility compared to major ministries with larger budgets, new evidence-based actions can be introduced such as literature reviews related to the sectorial preoccupations of the small-budget ministry. Network formation can happen whenever the lead ministry brings new partners to the table who can expand the network of policy implementers and designers. For example, when the lead ministry receives questions from a partner ministry about the state of the art in a new topic, it can respond by inviting an expert on the topic to present. Managing the innovation process can happen whenever continuous alignment and multi-actor coherence is encouraged among partner ministries, for instance, during yearly events at which professionals and managers from partner ministries present their opportunities and threats to implementing policy AB for the following year.

An additional function that organisations and implementation scholars might want to consider is trending know-how on EBP, namely moving beyond knowledge to incorporate consideration for exchange of such know-how and its capitalisation. A process might be more efficient and run more smoothly when the team can benefit from the experience of other colleagues who have faced similar challenges, namely the challenge of formulating a policy in a dispersed environment. Consider the stage when the budget for policy AB has to be written and the team experience intersectoral budget design for the first time. Here, they could benefit from exchanges about previous budget-related tools developed by partners, or from exchanges with previous intersectoral teams facing similar design steps.

Public organisations might be more efficient at building upon existing know-how and their own internal experience of which tools and practices function well. They might also be more efficient at feeding public administration with their own know-how, or practical experience, for the sake of (other) organisational (or governmental) learning curves. In addition to standard innovation-broker capacities, trending know-how on EBP appears relevant to organisations fostering EBP.

#### The policy broker’s role in designing EBP: capture unfulfilled needs, coordinate interest and convey evidence outward

We are all aware of think tanks and lobbyists pushing their agenda forward or even creating windows of opportunity. Such actors are either referred to as policy brokers or policy entrepreneurs [7]; herein, we use the term policy broker. A policy broker plays a pivotal role in the policy-making process, from agenda-setting to implementation. Indeed, a policy broker raises interest in and seeks approval of a new policy [8], spots changing policies, and tries to get interest groups or organisations to find solutions and add concerns to groups’/organisations’ agenda [7]. A policy broker supposedly helps to discover unfulfilled needs, suggests means, assembles and coordinates actors, and bears reputational risks. The policy broker is key to innovation and facilitation processes and advocates a more centrist position.

Policy brokers introduce, translate and help implement solutions, acting by choice rather than on command. A policy broker might exploit an opportunity and try to create beneficial situations without regard for resources currently not within their control. Finally, the main capacities that a policy broker acts in are to capture unfulfilled needs, to advocate for new ideas (a common capacity with the innovation broker), to translate these ideas into solutions that are empirically feasible (a common capacity with the knowledge broker), and to coordinate the spread of interest.

During the formulation of policy AB, these capacities could translate in the following ways. The lead ministry could capture unfulfilled needs by looking at discrepancies between what emerged from the commissioned literature review and the existing measures within its jurisdiction; as a result, policy AB then adds an extra measure. Additionally, to maintain interest in the importance of evidence and coordinate the spread of interest in EBP, the lead ministry of policy AB could put new management tools in place. Indeed, it faces the challenge that partner ministries will go back to their old habits, attending intersectorial meetings in a manner that reinforces one-way communication – from the lead ministry to its partners –and altogether disfavouring EBP in favour of reproducing and continuing existing measures and plans. To counteract this, the lead ministry could animate meetings on cutting-edge topics, invite experts to present new evidence, and so on.

The additional function that implementation scholars and organisations interested in EBP might want to consider is to convey evidence and solutions outward, towards policy partners and partner ministries, and within partner ministries. Indeed, traditionally, ministries’ communication style tends to focus on the information being sent from the ministry to the media or to the ministry’s websites, and to focus much less on how to communicate to partners and partner ministries. Those latest may not emphasise communication styles in general, and communication of evidence in particular in a similar fashion. During policy design, the coordination of the team/departmental/sectoral agenda and team/departmental/sectoral degree of action must be addressed.

While exchanges with some ministries can suggest new evidence-based measures to incorporate into the policy, in the end, such propositions are lost, watered down or dropped. For example, professionals from participating teams, departments or ministries might be present during discussions on policy AB on, for instance, Health in All Policies. However, when they return to their own teams, departments or ministries, they may have difficulty convincing their fellow public servants or in formulating the idea in a way their colleagues might understand and accept. When a participant’s colleagues do not take part in the original exchanges, the translation of evidence to them and the entire process of developing a shared understanding of knowledge and boundary knowledge is limited or impaired. As a result, discussions can at times become bogged down or altered within the partner teams, departments or ministries.

The lead organisation usually does not become involved, neither directly nor indirectly, in the vertical transfer within partners, arguably not having the authority to do so. The phenomenon here looks something akin to hearing things through the grapevine. To prevent the weakening of the message, the policy-broker role appears particularly important in ensuring that evidence and solutions are carried outside the lead organisation, deep into the structures involved in the design of an EBP.

### Conclusions

In this editorial, we propose consideration of the brokerage capacities that a modern public administration must own and foster to ensure a high appropriation of evidence by a dispersed group of actors (Box 1). We distinguish 11 brokering capacities, of which three are specific to the design of an EBP, namely (1) capturing boundary knowledge, (2) trending know-how on EBP across the actors involved, and (3) conveying evidence outward*,* towards partner ministries and within partner ministries*.* Such brokering capacities can feed into future analysis for scholars interested in EBP and organisational capacities.

The main contribution of this Commentary relates to its spotlight on know-how related to EBP. By know-how we mean the practical knowledge “*of how to do policy work*” [9]. Maybin mentioned that know-how in policy capacity is a key component for “*getting the right people on board*” [9]. Herein, we provide specific know-how on EBP for organisations. It is customary that brokering inputs atilt towards lobby-informed policy rather than evidence-informed policy. We intend to fill this gap. The EBP literature is criticised for its primary focus on “*academics’ research priorities over those of policymakers*” [10], lacking interactions and alignments between the producers and users of evidence. The focus on capacity for organisations to foster EBP is one attempt at providing reflections for policy-makers and direct designers of policy.

We also provide substance for developing measurements. We propose a combination of brokering capacities, which could help implementation scholars to think about and develop certain measurements to assess the relevance of actions and the readiness of organisations involved in EBP design. They might also be informative for health ministries who play the role of coordinators of policies, as well as for partners impacting determinants of health and involved in policies. Because little research has explored the specific capacity needed for a lead organisation, the brokering capacities we propose here could be a starting point.

## Box 1: Eleven organisational brokering capacities for the design of evidence-based policy

1. Transfer knowledge

2. Translate knowledge

3. Transform knowledge

4. Capture boundary knowledge

5. Articulating needs and demands

6. Forming network

7. Managing innovation

8. Trending know-how on evidence-based policy

9. Capture unfulfilled needs

10. Coordinate interests

11. Convey solutions and evidence outward

## Abbreviation

EBP: evidence-based policy
